# A Persistent Parvovirus Infection Causing Anemia in an HIV Patient Requiring Intravenous Immunoglobulin Maintenance Therapy

**DOI:** 10.7759/cureus.24627

**Published:** 2022-04-30

**Authors:** Dhairya Gor, Vinit Singh, Varsha Gupta, Michael Levitt

**Affiliations:** 1 Internal Medicine, Jersey Shore University Medical Center, Neptune, USA; 2 Internal Medicine, Monmouth Medical Center, Long Branch, USA; 3 Hematology and Oncology, Jersey Shore University Medical Center, Neptune, USA

**Keywords:** hematology, hiv, intravenous immunoglobulins, parvovirus b19, anemia

## Abstract

Anemia is a common finding in a human immunodeficiency virus (HIV)-positive patient with a wide range of possible causes and is a significant risk factor for mortality in acquired immunodeficiency syndrome (AIDS). Opportunistic parvovirus infection-causing pure red cell aplasia is one of its uncommon causes. It has been suggested that immunocompromised patients with abnormal antibody production are more susceptible to acquiring a chronic parvovirus infection requiring long-term intravenous immunoglobulin (IVIg) treatment; however, there are no specific guidelines for it. Here, we present a case of an HIV patient with persistent parvovirus infection resulting in chronic anemia requiring long-term maintenance immunoglobulin therapy with an excellent therapeutic response.

## Introduction

Parvovirus is a single-stranded DNA virus that gets its name from the Latin word "parvum," which means small [1]. It is mainly transmitted through respiratory droplets, although it can also be transmitted through other means such as blood products and organ transplants [1]. Acute parvovirus infection can cause transient aplastic crises and pure red cell aplasia with persistent infection by infecting the erythroid precursors in the bone marrow [2]. Patients with immunocompromised states, e.g., HIV-infected, undergoing chemotherapy or organ transplantation, are particularly at the risk of persistent and severe anemia secondary to parvovirus infections [3]. In addition, these patients are susceptible to infection relapses, highlighting the significance of ongoing monitoring and maintenance therapy. Intravenous immunoglobulin (IVIg) is an effective treatment for parvovirus-induced pure red cell aplasia; however, there are no specific treatment guidelines for chronic parvovirus infection [4]. We discuss the management and therapeutic response of a 38-year-old HIV-positive male with persistent parvovirus infection treated with monthly immunoglobulin therapy.

## Case presentation

A 38-year Hispanic male with a medical history of HIV and surgical history of splenectomy for pancytopenia around 10 years ago presented to the emergency room (ER) with severe fatigue and palpitations for one week. His initial laboratory findings (Table 1) showed a hemoglobin (Hb) level of 3.3 g/dl with normocytic normochromic red blood cells (RBCs). The patient's absolute reticulocyte count was 0.011 x 10^6^/μL, and the reticulocyte index was 0.08%. His baseline hemoglobin one year ago was 11 g/dl. He admitted to not taking his HIV medications for the last year. In the ER, he received blood transfusions.

**Table 1:** Summary of laboratory results on initial presentation LDH: Lactate dehydrogenase; PCR: Polymerase chain reaction; Ab: Antibody; Ig: Immunoglobulin; PV B19: Parvovirus B19.

Laboratory test (Feb 2020)	Results	Unit	Reference range
Hemoglobin	3.3	g/dl	13.2-17.5
Hematocrit	9.8	%	40-53
Mean corpuscular volume	83.1	fL	80-100
White blood cells	9,000	/µL	4,500-11,000
Platelets	587,000	/µL	140,000-400,000
LDH	119	U/L	91,000-200,000
Haptoglobin	173	mg/dL	30-225
Reticulocyte count	0.91	%	0.40-2.50
Absolute reticulocyte count	0.011 x 10⁶	/µL	0.010-0.110
HIV RNA PCR	1226	Copy/ml	None
Parvovirus DNA	Detected		None
Ab IgM PV B19	3.20		= 0.89
Ab IgG PV B19	1.59		= 0.89

The patient's workup was negative for opportunistic infections, including toxoplasma IgM, cryptococcal antigen (Ag), syphilis enzyme immunoassay (EIA), and acid-fast blood culture, and he had normal hemoglobin electrophoresis along with an unremarkable iron panel, vitamin B12, and folate levels. However, parvovirus B19 quantitative polymerase chain reaction (PCR) was >100,000,000 with peripheral blood smear showing atypical lymphocytosis, abnormal RBC morphology, target cells (1+), red cell distribution width of 15.3%, and CD4 count of 45 cells/mm^3^. Due to the patient's unwillingness, a bone marrow biopsy was not performed in our case, and there was no medication explaining the anemia.

Provisionally, the patient was diagnosed with chronic parvovirus infection with pure red cell aplasia causing severe anemia on initial admission and received IVIg of 2 g/kg total dose. He was also restarted on anti-retroviral medication, including an emtricitabine-rilpivirine-tenofovir combination of one tablet daily. By the time of discharge, his blood counts responded, and his Hb was stable at 7.2 g/dL; however, he was found to have persistent viral titers of parvovirus B19. After discharge, the patient did not follow up in the clinic. Unfortunately, the patient had a relapse about six months later and presented to the ER with a Hb of 4.8 g/dl and normocytic normochromic anemia with an absolute reticulocyte count of 0.060 x 10^6^/μL and reticulocyte index of 0.46%. He underwent endoscopy with findings of plaques in the esophagus but no active bleeding ulcers, lesions, or evidence of carcinoma. Relevant laboratory findings are shown in Table 2.

**Table 2:** Summary of laboratory results on repeat presentation LDH: Lactate dehydrogenase; CD4 Abs: Absolute cluster of differentiation 4; Ab: Antibody; Ig: Immunoglobulin; PV B19: Parvovirus B19.

Laboratory tests (Sep 2020)	Results	Unit	Reference range
Hemoglobin	4.8	g/dl	13.2-17.5
Hematocrit	13.9	%	40-53
Mean corpuscular volume	84.2	fL	80-100
White blood cells	5,500	/µL	4,500-11,000
Platelets	611,000	/µL	140,000-400,000
LDH	173	U/L	91,000-200,000
Haptoglobin	134	mg/dL	30-225
Reticulocyte count	2.48	%	0.40-2.50
Absolute reticulocyte count	0.060	/µL	0.010-0.110
CD4 Abs	45	cells/µL	430-1,800
Parvovirus DNA	>100,000,000	IU/mL	none
Ab IgM PV B19	0.49		= 0.89
Ab IgG PV B19	0.58		= 0.89

**Figure 1:**
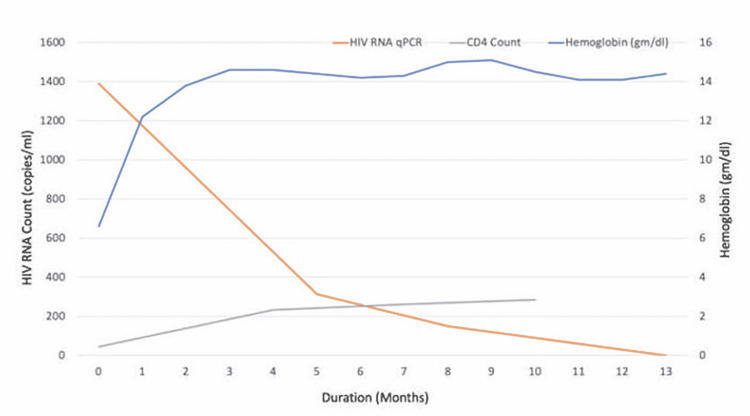
Graph showing the trend in hemoglobin levels, HIV RNA copies, and CD4 counts after treatment initiation CD4: Cluster of differentiation 4; qPCR: quantitative polymerase chain reaction.

**Figure 2:**
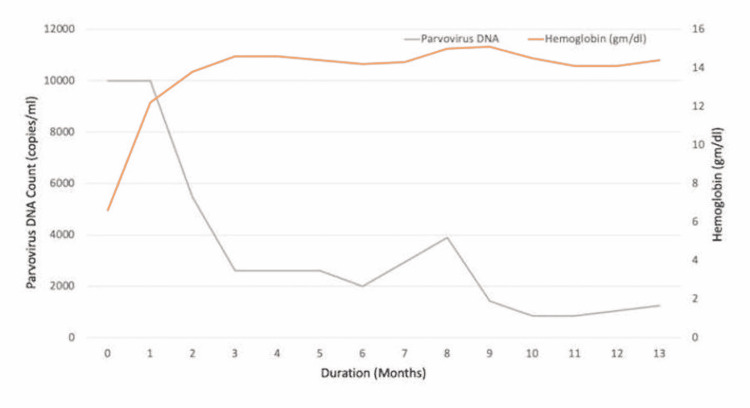
Graph showing the trend in hemoglobin levels and parvovirus DNA copies after treatment initiation

After multiple blood transfusions, his hemoglobin stabilized at 9.3 g/dL, and he was subsequently discharged. Upon follow-up in two weeks, the hemoglobin dropped again to 6.7 g/dL. He was then treated with IVIg 1 g/kg x two days and since then has been requiring monthly maintenance IVIg of 0.4 g/kg for the last year. While on maintenance IVIg, he has not required breakthrough blood transfusions or hospitalizations. He reported improved adherence to his highly active antiretroviral therapy (HAART) regimen, with gradual improvement in CD4 counts and decreasing viral load. Parvovirus levels on PCR decreased but continued to have detectable parvovirus levels suggesting chronic parvovirus infection. The last year's laboratory trend is shown in Figures 1, 2.

## Discussion

Parvovirus is associated with transient aplastic crises and pure red cell aplasia by infecting the erythroid precursors in the bone marrow [1,2]. Rapidly dividing pronormoblasts are most susceptible to the viral attack leading to its arrest at this stage [5]. Anemia usually resolves after the production of antibodies against the virus [4,6]. Patients with HIV or immunosuppressive conditions have a reduced ability to clear the parvovirus due to an inadequate antibody response, predisposing them to a higher risk of severe and chronic anemia [2,7]. Anemia in HIV patients should be adequately managed because it is a substantial risk factor for disease progression and death, regardless of CD4 cell counts or viral load [5,8]. It can be caused by various factors, including drug therapy such as zidovudine and didanosine, the virus itself, infiltrative disorders of bone marrow, and opportunistic infections [2].

Our patient's workup was consistent with parvovirus infection, as mentioned above. It is essential to remember that while antibodies may be undetectable in immunocompromised patients, virus levels are readily detectable [1]. Similarly, our patient with AIDS had undetectable IgM and IgG antibodies at presentation despite a very high viral load. Parvovirus B19 DNA is the most reliable method to demonstrate infection. The absence of erythroid progenitor cells and the presence of gigantic pronormoblasts characterize the bone marrow findings in persistent parvovirus infection [1]. However, bone marrow results are neither sensitive nor specific for identifying chronic B19 infections in HIV patients [5].

IVIg is an effective treatment for parvovirus-induced pure red cell aplasia [3,4]. The infused antibodies, which contain large amounts of anti-HPV-B19 IgG, can neutralize the virus. There are no explicit guidelines regarding the treatment of chronic parvovirus infection. However, there have been reports of patients receiving IVIg for several months for chronic parvovirus infection. For example, an HIV patient was treated with IVIg for severe refractory anemia for 32 days, totaling 175 g of IVIg [2]. Post-treatment viral levels were down to 1 ng/ml with an increase in IgG antibodies, reticulocyte count, and stabilization of hematocrit [2]. In another case, IVIg was given for around seven months until the viral load was undetectable in the blood or the bone marrow, and the hemoglobin returned to normal levels [9].

A study of eight AIDS patients with persistent pure red cell aplasia was conducted between 1993 and 1997 to determine the effects of IVIg, with an initial treatment of 1 g/kg per day for the first one to two days of treatment [4]. Six patients with a CD4 count of fewer than 80 cells/mm^3^ who received 1 g/kg IVIg for two days had disease relapse [4]. Second and subsequent relapses were treated with IVIg 0.4-1 g/kg every four weeks and monitored for a mean period of 27 months [4]. In all patients with CD4 counts below 80 cells/mm^3^, it was proposed that routine maintenance therapy with IVIg 0.4 g/kg every four weeks be considered [4].

In our case, the patient received an IVIg total of 2 g/kg on initial presentation and relapse. It was followed by maintenance therapy of IVIg 0.4 g/kg every four weeks for the last 11 months. We monitored the viral load, hemoglobin levels, and CD4 cell count monthly to see how the treatment responded. Figures 1 and 2 illustrate the values. The patient was maintained on IVIg every four weeks due to persistent viremia. Treatment of underlying HIV is of paramount importance to fight the chronic persistent infection [10,11]. The usefulness of IVIg maintenance therapy in avoiding relapses has not been thoroughly investigated [6]. This case highlights the challenging management of refractory parvovirus disease.

## Conclusions

Patients with HIV are at the risk of developing persistent parvovirus infection leading to chronic anemia. The use of long-term maintenance IVIg provides a favorable therapeutic outcome. However, it is challenging to determine the optimal dose and duration of IVIg treatment for chronic parvovirus infection to minimize relapses and recurring hospitalizations. We have been treating our patient for around one year with IVIg who had a good response, and we believe reporting our findings may aid in the management of similar cases. To define treatment guidelines further, prospective studies analyzing the effectiveness of chronic maintenance therapy should be conducted.
